# Antimicrobial Activity of Selected Essential Oils against Selected Pathogenic Bacteria: In Vitro Study

**DOI:** 10.3390/antibiotics10050546

**Published:** 2021-05-08

**Authors:** Nikola Puvača, Jovana Milenković, Tamara Galonja Coghill, Vojislava Bursić, Aleksandra Petrović, Snežana Tanasković, Miloš Pelić, Dragana Ljubojević Pelić, Tatjana Miljković

**Affiliations:** 1 Faculty of Biomedical and Health Sciences, Jaume I University, Avinguda de Vicent Sos Baynat, s/n, 12071 Castelló de la Plana, Spain; 2 Department of Engineering Management in Biotechnology, Faculty of Economics and Engineering Management in Novi Sad, University Business Academy in Novi Sad, Cvećarska 2, 21000 Novi Sad, Serbia; galonja@fimek.edu.rs; 3 Faculty of Pharmacy, University of Belgrade, Vojvode Stepe 450, 11221 Belgrade, Serbia; jovana.milenkovicbg1987@gmail.com; 4 Department for Phytomedicine and Environmental Protection, Faculty of Agriculture, University of Novi Sad, Trg Dositeja Obradovića 8, 21000 Novi Sad, Serbia; bursicv@polj.uns.ac.rs (V.B.); aleksandra.petrovic@polj.uns.ac.rs (A.P.); 5 Faculty of Agronomy in Čačak, University of Kragujevac, Cara Dušana 34, 32102 Čačak, Serbia; stanasko@kg.ac.rs; 6 Scientific Veterinary Institute Novi Sad, Rumenački put 20, 21000 Novi Sad, Serbia; milosp@niv.ns.ac.rs (M.P.); dragana@niv.ns.ac.rs (D.L.P.); 7 Faculty of Medicine, University of Novi Sad, Hajduk Veljkova 3, 21000 Novi Sad, Serbia; tatjana.miljkovic@mf.uns.ac.rs

**Keywords:** antibiotic resistance, microbes, essential oils, *E. coli*, *S. aureus*, *S. Thypi*, *C. koseri*

## Abstract

The worldwide problem of infectious diseases has appeared in recent years, and antimicrobial agents are crucial in reducing disease emergence. Nevertheless, the development and distribution of multidrug-resistant (MDR) strains in pathogenic bacteria, such as *Escherichia coli*, *Staphylococcus aureus*, *Salmonella Typhi* and *Citrobacter koseri*, has become a major society health hazard. Essential oils could serve as a promising tool as a natural drug in fighting the problem with these bacteria. The current study aimed to investigate the antimicrobial effectiveness of tea tree (*Melaleuca alternifolia* (Maiden and Betche) Cheel), rosemary (*Rosmarinus officinalis* L.), eucalyptus (*Eucalyptus obliqua* L’Hér.), and lavender (*Lavandula angustifolia* Mill) essential oils. The antimicrobial properties of essential oils were screened against four pathogenic bacteria, *E. coli*, *S. aureus*, *S. Tyhpi,* and *C. koseri*, and two reference bacterial strains, while for the testing, the agar well diffusion method was used. Gas chromatography (GC) and gas chromatography–mass spectrometric (GC–MSD) analyses were performed on essential oils. The obtained results showed that *M. alternifolia* essential oil is the richest in terpinen-4-ol, *R. officinalis* and *E. oblique* essential oils in 1,8-cineole, and *L. angustifolia* essential oil in α-terpinyl acetate. In addition, the main bioactive compounds present in the essential oil of tea tree are rich in α-pinene (18.38%), limonene (7.55%) and γ-terpinene (14.01%). The essential oil of rosemary is rich in α-pinene (8.38%) and limonene (11.86%); eucalyptus essential oil has significant concentrations of α-pinene (12.60%), *p*-cymene (3.24%), limonene (3.87%), and γ-terpinene (7.37%), while the essential oil of lavender is rich in linalool (10.71%), linalool acetate (9.60%), α-terpinyl acetate (10.93%), and carbitol (13.05%) bioactive compounds, respectively. The obtained results from the in vitro study revealed that most of the essential oils exhibited antimicrobial properties. Among the tested essential oils, tea tree was discovered to demonstrate the strongest antimicrobial activity. The recorded MIC of *S. Typhi* was 6.2 mg/mL, 3.4 mg/mL of *C. koseri*, 3.1 mg/mL of *E. coli*, and 2.7 mg/mL of *E. coli* ATCC 25922, compared to *M. alternifolia*. Similarly, only *S. aureus* ATCC 25923 showed antimicrobial activity towards *R. officinalis* (1.4 mg/mL), *E. oblique* (2.9 mg/mL), and *L. angustifolia* (2.1 mg/mL). Based on the obtained results, it is possible to conclude that tea tree essential oil might be used as an ecological antimicrobial in treating infectious diseases caused by the tested pathogens.

## 1. Introduction

The worldwide dispersion of resistant clinical isolates has led to the necessity to discover new antimicrobial agents [1]. Nevertheless, the earlier record of the precipitous, prevalent resistance to freshly created antimicrobial agents suggests that new families of antimicrobial agents will also have a short lifespan [2,3,4,5]. Many aromatic and medicinal plants, herbs, and spices have been proposed as a significant source of natural antimicrobials as an alternative to synthetic drugs to treat bacterial infections [6]. Medicinal plants and the essential oil extracted from them due to the high concentration of bioactive compounds have been widely used for this purpose [7,8,9]. It has been proven that essential oils have been used to treat urinary tract infectious diseases [10], respiratory diseases [11], intestinal disorders [12], and dermal illnesses [13].

Tea tree (*Melaleuca alternifolia* (Maiden and Betche) Cheel), rosemary (*Rosmarinus officinalis*), eucalyptus (*Eucalyptus obliqua* L’Hér.), and lavender (*Lavandula angustifolia* Mill) are aromatic and medicinal plants that belong to two different botanical families. With industrial development, especially in the past twenty years, large efforts have been made to identify and quantify these plants’ phenolic components [9,14]. The essential oils of these plants are rich in thymol, carvacrol, *p*-cymene, and γ-terpinene [15]. A series of studies have shown the positive effect of essential oils and their bioactive compounds thymol and carvacrol due to several biological properties: antioxidant [16], antimicrobial [17], antiviral [18], diaphoretic [19], expectorant [20], insecticidal [21], and genotoxic [22]. Due to their typical aroma and proximate composition, tea tree, rosemary, eucalyptus, lavender, are commonly utilized in agriculture, pharmaceutical, cosmetic, and food industries, respectively.

Research on extracts of both Myrtle and Lamiaceae family plant chemicals has investigated their composition and their other beneficial properties in in vitro and in vivo experiments [23,24]. As they are secondary plant metabolites, the concentration is influenced by genetic and paragenetic factors, so the constant investigation and determination of their concentrations in plants are of high importance [25].

In recent decades, *E. coli* and *S. aureus* have accounted for the most significant number of outbreaks, cases, and deaths worldwide [1]. To decrease health hazards and economic losses due to the emergence of these pathogens, the use of natural antibacterial alternatives seems to be an appealing way to control the incidence of pathogenic bacteria [26].

*Salmonella Tyhpi* is most often the cause of typhoid fever, which is a profoundly severe intrusive bacterial disease of humans. *S. Tyhpi* can aggressively colonize the mucosal surface of the humane digestive tract but are generally confined in healthy people by the local immune defense mechanisms. Still, *S. Typhi* has developed the capability to propagate to deeper tissues, such as the bone marrow, spleen, and liver [27]. A distinctive characteristic of *Citrobacter koseri* is the exceedingly elevated tendency to initiate brain abscesses in neonatal meningitis. Earlier reports and studies on infant rats have documented many *Citrobacter*-filled macrophages within the ventricles and brain abscesses. It has been hypothesized that intracellular survival and replication within macrophages may be a mechanism by which *C. koseri* subverts the host response and elicits chronic infection, resulting in brain abscess formation [28].

Contemplating the considerable capability of essential oils as sources for natural antimicrobial drugs, this study aimed to investigate the antimicrobial effectiveness of tea tree, rosemary, eucalyptus, and lavender essential oils against pathogenic bacteria *E. coli*, *S. aureus*, *S. Tyhpi*, and *C. koseri* in in vitro conditions.

## 2. Results and Discussion

Bioactive substances are types of chemicals found in small amounts in plants and certain food (such as fruits, vegetables, nuts, oils, and whole grains). Actions in the body that are provided by bioactive compounds may promote good health [29]. They have been studied in the prevention of cancer, heart disease, and other diseases [30,31]. Different subgroups, including phenolic acids, flavonoids, tannins, coumarins, lignans, quinones, stilbenes, and curcuminoids, may be segregated by their chemical structures [32]. The results shown in Table 1 present the most dominant subgroup of the bioactive compound of each investigated essential oil.

**Figure 1:**
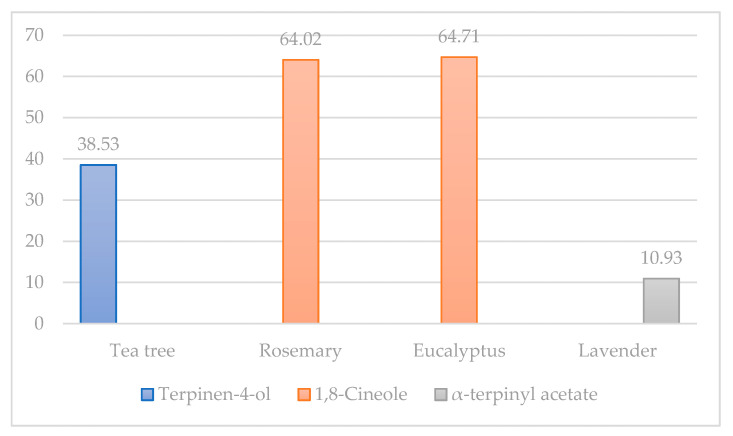
The highest concentrations of bioactive compounds in analyzed essential oils, %.

Conducted analyses show that the tea tree essential oil is richest in terpinen-4-ol, rosemary and eucalyptus essential oils in 1,8-cineole, and lavender essential oil in α-terpinyl acetate, respectively (Figure 1). Nevertheless, investigated essential oils in our research came with a declaration of origin, but the lack of regulation of the chemical composition of essential oils and the growing popularity of these oils among consumers present an urgent need for the accurate characterization of various oil types from a variety of manufacturers. Many essential oils in retail stores contain chemical substances of adulterants with potential toxicity [34]. In addition to the main bioactive compounds, the results of our research showed that the essential oil of tea tree is rich in α-pinene (18.38%), limonene (7.55%), and γ-terpinene (14.01%), respectively. Obtained results showed that rosemary essential oil was rich in α-pinene (8.38%) and limonene (11.86%); eucalyptus was rich in α-pinene (12.60%), *p*-cymene (3.24%), limonene (3.87%), and γ-terpinene (7.37%); and lavender was rich in linalool (10.71%), carbitol (13.05%), linalool acetate (9.60%), and α-terpinyl acetate (10.93%), respectively.

The two most popular essential oils on the market are tea tree and lavender oil [34]. Dubnicka et al. [34] investigated the adulteration of essential oils, which showed that six store brand essential oils, tea tree, lavender, sandalwood, rose, eucalyptus, and lemongrass, contained carbitol in concentrations from 23% to 35%, and four of the six oils contained diethyl phthalate in concentrations ranging from 0.33% to 16%. These toxicants are particularly concerning because they are known inhalation hazards, and the intended usage of these oils is for aromatherapy [34]. Based on our results and the high concentration of carbitol (13.05%) in lavender essential oil, we can assume that our lavender essential oil was not natural, which was revealed by the high concentration of carbitol as the contaminant and should be pointed out as a possible threat. Alpha-pinene presents a polyphenolic terpene organic compound [35]. It has been reported that α-pinene is a strong antioxidant agent which inhibits prostaglandin E1 and NF-κB and thus contributes to its anti-inflammatory and anticarcinogenic effects. Terpene is a part of many medical, aromatic, and spice plants [36]. Research has shown that limonene is usually found in oils obtained from citrus plants, but it has also been found in cannabis. Limonene is used to performed the percutaneous transfer of medicines in vitro and in vivo [37]. Gamma-terpinene is one of four isomeric monoterpenes. It is a naturally occurring terpenine and has been isolated from many different botanical sources [38]. It has the highest boiling point of the four known terpnine isomers (α-terpinine, β-terpinene, and δ-terpinine). It is a major component of various essential oils and has strong antioxidant activity [39,40]. It has a lemon-like or lime-like odor that is most commonly used in the food, aroma, soap, cosmetics, pharmaceuticals, tobacco, clothing and perfume industries.

**Figure 2:**
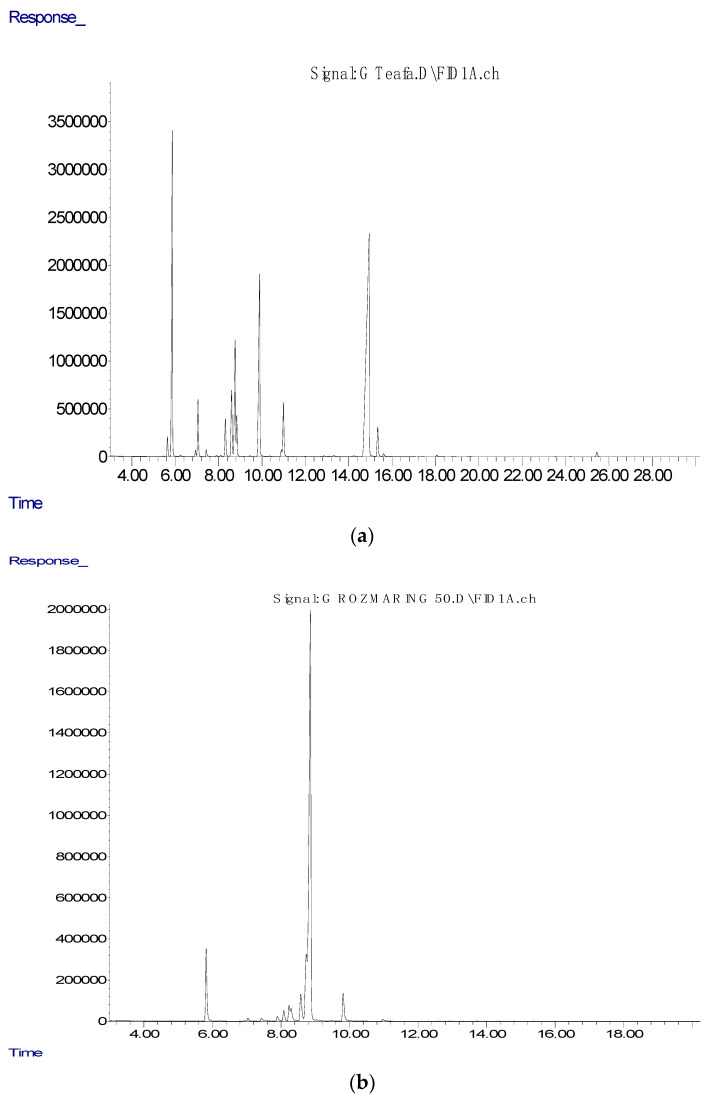

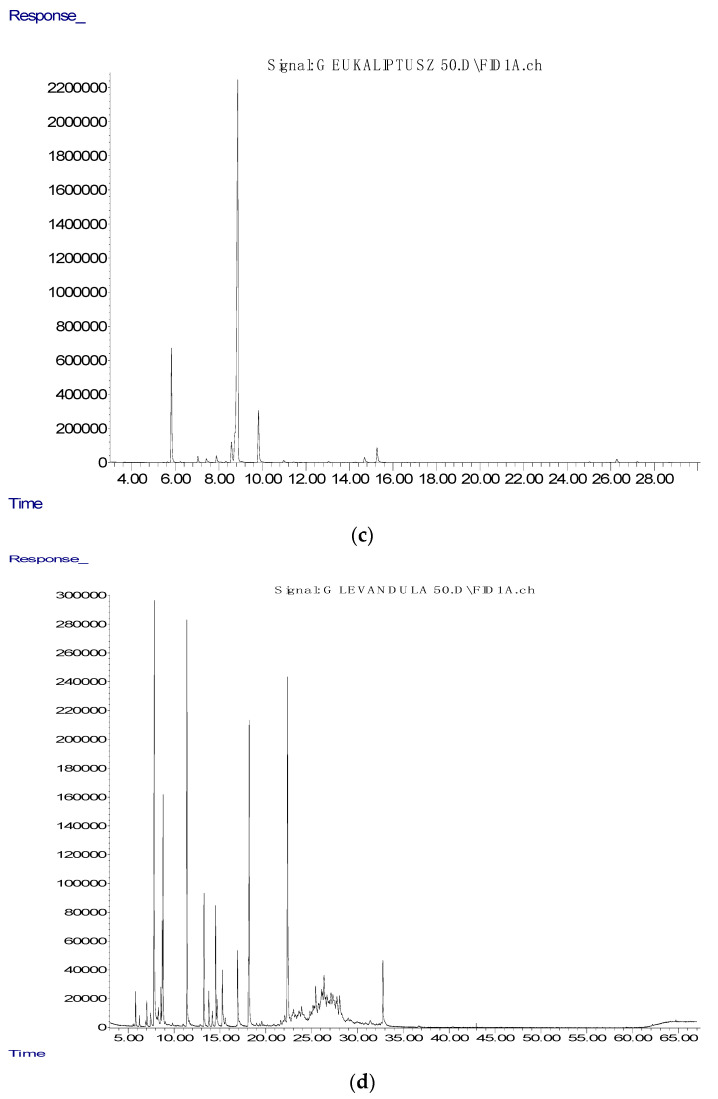
The peaks of chromatography analysis of tea tree (**a**), rosemary (**b**), eucalyptus (**c**), and lavender (**d**) essential oils.

Many experiments have shown the positive influence of these bioactive compounds found in essential oils, which was the topic of our research. Hendel et al. [31] in their research analyzed essential oils from the aerial parts of 15 samples of Algerian rosemary. The GC-MSD, as in our study, for the determination of phenolic compound was used. Thirty-eight components were characterized, with the highest share of α-pinene, camphene, and limonene as the main components; camphor, 1,8-cineole, and borneol as the principal oxygenated substances; caryophyllene, α-bisabolol, and humulene as the most represented sesquiterpenes. Furthermore, Hendel et al. [41] evaluated essential oils for their antimicrobial activity against *E. coli* and *S. aureus* and against ten fungal strains belonging to Aspergillus *Alternaria*, *Candida*, *Fusarium*, *Penicillium*, and *Saccharomyces* species, where the results showed moderate antimicrobial activity. Our results showed that eucalyptus essential oil is richest in eucalyptol (1,8-cineole), (Figure 1), while significant concentrations of α-pinene (12.60%), *p*-cymene (3.24%), limonene (3.87%), and γ-terpinene (7.37%) were reordered, respectively (Table 1). Eucalyptus essential oil, as well as rosemary, poses numerous beneficial properties. For example, phenolic compounds, such as camphene, α-pinene, and 2-phenyl ethanol, have high insecticidal properties of eucalyptus essential oil, so they present a potential candidate for application in integrated pest management approaches [42]. Reyes et al. [33] confirmed the fumigant and repellent action of eucalyptus essential oil against *Hypothenemus hampei*. The toxic effect of eucalyptus essential oil on the coffee berry borer is due to a synergistic effect involving 1,8-cineole, α-pinene, and *p*-cymene, according to investigations of Reyes et al. [43]. Results of our study showed that the essential oil of lavender was rich in linalool (10.71%), linalool acetate (9.60%), and α-terpinyl acetate (10.93%) bioactive compounds, respectively. Additionally, we found a significant concentration of carbitol (13.05%) in investigated lavender essential oil, which is a particular indication of essential oil adulteration. Our assumptions have also been confirmed by another study [34]. In addition to the bioactive compounds that we isolated from lavender essential oil in our research, Yadikar et al. [44] reported results that indicate isolations of seven new bioactive compounds from lavender. The same authors reported that they isolated lavandunat, lavandufurandiol, lavandufluoren, lavandupyrones A and B, and lavandudiphenyls A and B, along with five known compounds, benzoic acid, methyl propanoate, rosmarinic acid, and isosalvianolic acid C, from the ethyl acetate extract of the remaining material, which was obtained from lavender essential oil. According to the research of Sen et al. [45], in addition to the aforementioned essential oils, stated that the most produced peppermint essential oil in the Indian market also often has a high concentration of carbitol, which indicates adulteration. We also come to the same conclusion regarding the usage of lavender essential oil in our study. Donadu et al. [46] investigated the in vitro activity of lavender essential oil against drug-resistant strains of *P. aeruginosa*. Bearing in mind that lavender essential oil has been used for its anti-inflammatory, antidepressant, antiseptic, antifungal, and antimicrobial properties, the positive result in this research was expected. Donadu et al. [35] showed that lavender essential oil did not possess a cytotoxic effect when administered in very low concentration, while the same essential oil significantly reduced nitric oxide synthase activity on murine macrophages, which was also evaluated. Increased drug resistance and the absence of new antibiotics can promote the production of natural antimicrobial replacements, which is in agreement with numerous investigations of Puvača et al. [47]. Figure 2 presents the peaks of chromatography analysis of the essential oils of tea tree (a), rosemary (b), eucalyptus (c), and lavender (d) used in this research.

The bioactive compounds of essential oils were tentatively identified (Table 1). All investigated essential oils in our research with their main components exhibit a broad spectrum of antimicrobial activity, which can be principally attributed to terpinen-4-ol (tea tree), 1,8-cineole (rosemary and eucalyptus), and carbitol (lavender), as active substances (Figure 1).

All worldwide countries, developed or developing, are equally affected by antibiotic resistance [48]. The development and distributions of MDR pathogens have significantly compromised the present antibacterial therapy [49]. This emergence and antibiotic resistance emergence have led to a search for new antimicrobial substances of natural origin. Essential oils are known to be rich in bioactive compounds with numerous curative properties [50]. Our research was performed to investigate four different essential oils’ antimicrobial activities, with different main bioactive compounds compared to human pathogens and two reference bacterial strains.

The assessment of the antimicrobial activity in essential oils used in our study was determined by the disc diffusion method compared to *E. coli*, *S. aureus*, *S. Thypi*, and *C. koseri*. The tested pathogenic bacteria are repeatedly implicated in the occurrence of many diseases [51]. Our study showed that all essential oils that were used displayed a differing level of antimicrobial activity compared to pathogenic bacteria (Table 2).

The obtained results also revealed that the tea tree essential oil was the most useful among all the tested essential oils. The recorded zone of inhibition against *E. coli* was 21 mm, and against reference strain *E. coli* ATCC 25922 18 mm, 15 mm against *S. Typhi*, and 13 mm against *C. koseri*, respectively, while antimicrobial activity against *S. aureus* was not recorded. Other essential oils used in our study, rosemary, eucalyptus, and lavender, exhibited their antimicrobial activity against *S. aureus* and its reference strain with a zone of inhibition of 13 mm (Table 2), and *S. Typhi* with 15 mm, without any antimicrobial activity towards *E. coli* or its strain, or towards *C. koseri*.

The antimicrobial efficiency of essential oils was determined by measuring the minimum inhibitory concentration (MIC), as shown in Table 3. Among the tested essential oils in our study, tea tree was discovered to demonstrate strong antimicrobial activity. The recorded MIC of *S. Typhi* was 6.2 mg/mL, 3.4 mg/mL of *C. koseri*, 3.1 mg/mL of *E. coli*, and 2.7 mg/mL of *E. coli* ATCC 25922, compared to tea tree. Similarly, only *S. aureus* ATCC 25923 showed antimicrobial activity towards rosemary (1.4 mg/mL), eucalyptus (2.9 mg/mL), and lavender (2.1 mg/mL).

While tea tree essential oil showed a good antibacterial activity in nearly all bacterial isolates and strains of *E. coli* and *S. aureus*, other essential oils used in our study showed a constrained antibacterial activity contrary to the test bacterial isolates according to the obtained MIC values. Our result was similar to other findings that have reported antibacterial activity [52,53,54,55,56].

More stringent criteria regarding the activity were described by Saraiva [57] and Silva et al. [58], which specifically indicated that when MIC values < 100 μg/mL have been recorded, activity is described as high; when the obtained values are between 100 and 500 μg/mL, it is considered active; for those between 500 and 1000 μg/mL, activity is described as moderately active; for those between 1000 and 2000 μg/mL, activity is described as low; and those with MIC > 2000 μg/mL are described as inactive.

If taking into account the previously stated results, the effect observed in this study could be considered inactive (except for the effect of rosemary on *S. aureus*, where 1.4 mg/mL may be considered of low activity).

The in vitro antibacterial and antifungal activities of tea tree oil were investigated, and MICs for sixteen different microorganisms were determined by applying the broth dilution method. Tea tree oil showed the best overall antimicrobial effect [59]. The antimicrobial activity of tea tree essential oil has been known for a long time. Li et al. [52] investigated the dynamics and mechanism of its antimicrobial activities of tea tree essential oil in two bacterial strains. Poisoned food technique assessment showed that the MICs of tea tree essential oil for *E. coli* and *S. aureus* were 1.08 and 2.17 mg/mL, respectively. Antimicrobial dynamic curves showed that with increasing concentrations of essential oil, the rate of cell killing and the duration of the growth lag phase increased correspondingly [52]. The essential oil of tea tree exhibited a broad spectrum of antimicrobial activity. Its mode of action against the Gram-negative bacterium *E. coli* and the Gram-positive bacterium *S. aureus* was investigated using various methods. It has been reported that the exposure of these organisms to minimum inhibitory concentrations of tea tree oil inhibit respiration and increase the permeability of bacterial cytoplasmic and yeast plasma membranes [60].

**Figure 3:**
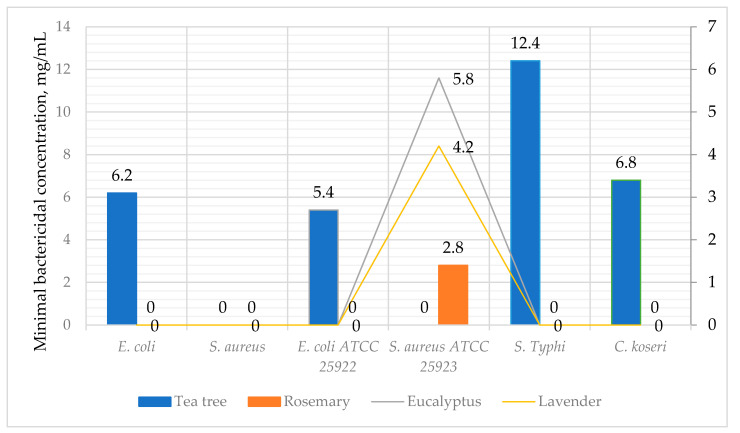
Minimal bactericidal concentration (MBC); values of essential oils against bacteria (mg/mL). Values expressed the MBC as >the maximum concentration tested (50 mg/mL).

The antimicrobial efficiency of essential oils was also determined by measuring the minimal bactericidal concentration (MBC), which is shown in Figure 3. Results of the MBC show that tea tree demonstrated the strongest antimicrobial activity. The recorded MBC of *S. Typhi* was 12.4 mg/mL, 6.8 mg/mL of *C. koseri*, 6.2 mg/mL of *E. coli*, and 5.4 mg/mL of *E. coli* ATCC 25922, compared to tea tree. Exceptionally, *S. aureus* ATCC 25923 showed bactericidal activity towards rosemary of 2.8 mg/mL, eucalyptus of 5.8 mg/mL, and lavender of 4.1 mg/mL.

Mohsen et al. [61] performed a study to evaluate the antimicrobial activity of rosemary essential oil human pathogenic bacteria. *E. coli* and *S. aureus* were selected for investigation, as well as three other bacteria. The antimicrobial activity of in vitro conditions showed that based on the disc diffusion agar method, the inhibition zone diameter of rosemary essential oil for *E. coli* was 12.10 mm. Authors concluded that rosemary essential oil is a suitable antibacterial agent and can be used as a natural alternative in the control of pathogenic microorganism growth [61].

Elaissi et al. [62] investigated the antibacterial activity of several *Eucalyptus* species and their correlation with chemical composition. The main chemical compounds were determined to be 1,8-cineole, spathulenol, *α*-pinene, *p*-cymene, and limonene. The most potent antibacterial activity was recorded against *S. aureus* and *E. coli*, while the correlation between the levels of active compounds in essential oil and the antibacterial activities was noticed. Similar results, which are in accordance with our findings, were demonstrated in the study of Vaghasiya and Chanda [63]. Authors investigated the antimicrobial and antifungal properties of eucalyptus essential oil and concluded that the most susceptible bacterium was *Citrobacter freundii*, while the most resistant was *Proteus vulgaris*.

Unfortunately, the antimicrobial properties of eucalyptus essential oil are very limited, but lavender essential oil and its effects in various fields have been investigated. Adaszyńska-Skwirzyńska and Szczerbińska [64] investigated the antimicrobial activity of lavender essential oil and its influence on the production performance of broiler chickens. Researchers concluded that the addition of 0.4 mL/L to the drinking water of broiler chickens had significantly improved production results, with a proven significant effect on bacterial growth inhibition. Another study was performed to verify the antimicrobial activity of lavender essential oil as the component of a preservative system in oil in water body milk [65]. The obtained results showed a reduction in bacteria in the inoculum by 3 logarithmic units within 7 days with no increase up to the 28th day. Bosnić et al. [66] investigated the antimicrobial activity of sage, rosemary, eucalyptus, melissa, lavender, and thyme essential oils against Gram-positive and Gram-negative bacteria. Based on their findings, it was concluded that the most active essential oils were eucalyptus and rosemary, with the MICs ranging from 0.097 to 0.390 mg/mL. The results of Shirugumbi Hanamanthagouda et al. [67] confirmed that lavender essential oil was inhibitory against various bacterial and fungal strains, respectively.

Although a certain number of essential oils show good antibacterial activity, some oils’ narrow antibacterial activities do not provide a complete picture for the usage of essential oil against the occurrence of infectious diseases. Nevertheless, further study is necessary to investigate their efficacy in inhibiting the growth of bacteria, fungi, parasites, and viruses.

## 3. Material and Methods

Commercially available essential oils of tea tree, rosemary, eucalyptus, and lavender used in this research were purchased from a local distributor in Novi Sad, Serbia. According to certification, essential oils from plants were extracted using supercritical CO_2_ in the conventional semi-continuous method to separate 1,8-cineole, linalool, linalyl acetate, and camphor.

Gas chromatography (GC) and gas chromatography–mass spectrometric (GC–MS) analyses were performed using an Agilent 7890A GC equipped with an inert 5975C XL EI/CI mass spectrometer detector (MSD) and flame ionization detector (FID) connected by a capillary flow technology 2-way splitter with make-up. The HP-5MS capillary column (30 m × 0.25 mm × 0.25 μm) was used. The GC oven temperature was programmed from 60 to 300 °C at a rate of 3 °C min^−1^ and held for 15 min. Helium was used as the carrier gas at 16.255 psi (constant pressure mode). An auto-injection system (Agilent 7683B Series Injector - Agilent Technologies Inc, Santa Clara, CA, USA) was employed to inject 1 μL of sample. The sample was analyzed in the splitless mode. The injector temperature was 300 °C and the detector temperature 300 °C. MS data were acquired in the EI mode with a scan range of 30–550 m/z, source temperature of 230 °C, and quadruple temperature of 150 °C; the solvent delay was 3 min.

The identification of all compounds in the analyses was matched by comparing their linear retention indices (relative to C8–C36 n-alkanes on the HP-5MSI column) and MS spectra with those of authentic standards from NIST11 databases.

Previously used structural, physical, and standard biochemistry assessments were used to pinpoint bacterial strains, followed by an antimicrobial susceptibility test by a modified Kirby Bauer disc diffusion method and the Clinical and Laboratory Standards Institute guidelines. Resistant isolates were identified as the isolates resistant to amikacin antibiotic.

A total of six human pathogenic strains were used in this study. All Gram-positive organisms were identified by conventional methods, such as Gram stain, positive catalase, tube coagulase, deoxyribonucleases (DNAse) tests. An API 20E kit was used to identify the Gram-negative organism.

The agar well diffusion method in Mueller-Hinton agar plates was used for antimicrobial testing of essential oils. Incubation of inoculated bacteria was conducted for 12 h at a temperature of 37 °C, in Nutrient broth. A Mueller-Hinton agar plate was cultured with standardized microbial culture broth. Essential oils in concentrations of 50 mg/mL were prepared in organosulfur solvent ((CH_3_)_2_SO). Four wells of 8 mm were bored in the inoculated media. Each well was filled with 50 μL of essential oils: positive control of amikacin (30 mcg) and nitrofurantoin (300 mcg) and negative control. The diffusion process lasted for about 30 min at a temperature of 22.5 °C and incubation time for 18–24 h at 37 °C. Following incubation, plates were examined to develop a clear zone around the well which corresponded to the antimicrobial activity. The zone of inhibition was detected and assessed (mm).

The broth microdilution method was used to establish the minimal inhibitory concentrations corresponding to the Clinical and Laboratory Standards Institute guidelines. Twin successive dilutions of essential oils were conducted directly in a microtiter plate containing Mueller-Hinton broth. The bacterial inoculum was added to 5 × 105 CFU/mL in each well. An antibiotic amikacin was used for the control reference. Incubation of plates was performed at temperature of 37 °C for 24 h. Resazurin was added to each well of the microtiter plate and incubated at 37 °C for 30 min. The occurrence of pink color was associated with wells which displayed bacterial growth, while the blue color was associated with those without bacterial growth. The minimal inhibitory concentrations were considered as the lowest concentration of the essential oil that completely inhibits bacterial growth.

## 4. Conclusions

Based on the obtained results, it can be concluded that tea tree essential oil is richest in terpinen-4-ol, rosemary, and eucalyptus essential oils in 1,8-cineole, and lavender essential oil in α-terpinyl acetate. In addition to the main bioactive compounds, the results of our research showed that the essential oil of tea tree is rich in α-pinene (18.38%), limonene (7.55%), and γ-terpinene (14.01%). The essential oil of rosemary is rich in α-pinene (8.38%) and limonene (11.86%); eucalyptus essential oil has significant concentrations of α-pinene (12.60%), *p*-cymene (3.24%), limonene (3.87%), and γ-terpinene (7.37%), while the essential oil of lavender is rich in linalool (10.71%), linalool acetate (9.60%), and α-terpinyl acetate (10.93%), respectively. It has also been found that lavender essential oil is rich in carbitol (13.05%) as a potentially toxic compound.

Our research showed tea tree essential oil’s antimicrobial activity towards *E. coli*, *S. Typhi*, and *C. koseri*, while the other essential oils exhibited their antimicrobial activity towards *S. aureus*. Although results showed some potential in the in vitro activity of investigated essential oils for pathogenic bacteria, these obtained results still may not be applied in vivo. Based on our in vitro findings, further research in in vivo conditions is necessary to evaluate the antimicrobial activity of investigated essential oils fully.

## Figures and Tables

**Table 1:** Identified bioactive compounds of analyzed essential oils, % ± SD.

Compound	Retention Indices	Retention Indices NIST ^1^	Retention Time	Tea Tree	Rosemary	Eucalyptus	Lavender
α-Thujene	922	924	5.636	1.10 ± 0.01	0.03 ± 0.00	0.06 ± 0.00	0.05 ± 0.01
α-Pinene	930	932	5.862	18.38 ± 0.08	8.38 ± 0.02	12.60 ± 0.01	0.72 ± 0.00
Camphene	945	946	6.241	0.08 ± 0.00	0.03 ± 0.00	0.10 ± 0.00	0.25 ± 0.01
Thuja-2,4(10)-diene	950	952	6.378			0.01 ± 0.00	
Sabinene	970	969	6.932	0.35 ± 0.01			0.12 ± 0.00
β-Pinene	974	974	7.047	3.19 ± 0.01	0.38 ± 0.01	0.84 ± 0.01	0.60 ± 0.02
Myrcene	988	988	7.428	0.45 ± 0.00	0.49 ± 0.00	0.58 ± 0.01	0.56 ± 0.01
Carbitol	1003	1001	7.863				13.05 ± 0.04
α-Phellandrene	1004	1002	7.9	0.09 ± 0.00	0.68 ± 0.00	0.94 ± 0.00	
Δ3-Carene	1009	1008	8.098	0.09 ± 0.00	1.45 ± 0.03	0.05 ± 0.01	
Hexyl acetate	1011	1009	8.146				0.13 ± 0.01
1,4-Cineole	1013	1012	8.235				
α-Terpinene	1015	1014	8.311	2.35 ± 0.01	2.02 ± 0.01	0.15 ± 0.00	0.41 ± 0.00
*p*-Cymene	1023	1020	8.598	4.30 ± 0.01	4.30 ± 0.05	3.24 ± 0.00	0.87 ± 0.01
Limonene	1027	1024	8.758	7.55 ± 0.01	11.86 ± 0.01	3.87 ± 0.01	2.23 ± 0.02
1,8-Cineole	1033	1026	8.864	2.15 ± 0.05	64.02 ± 0.04	64.71 ± 0.04	5.55 ± 0.01
(Z)-β-ocimene	1035	1032	9.035			0.28 ± 0.00	0.06 ± 0.00
β-(E)-Ocimene	1046	1046	9.45	0.08 ± 0.00	0.11 ± 0.00	0.02 ± 0.00	
γ-Terpinene	1058	1054	9.89	14.01 ± 0.01	4.06 ± 0.00	7.37 ± 0.00	0.05 ± 0.00
*p*-Mentha-2,4(8)-diene	1085	1083	10.891	0.38 ± 0.01			
Terpinolene	1088	1086	10.991	3.56 ± 0.02	0.31 ± 0.00	0.35 ± 0.00	0.04 ± 0.00
Linalool	1099	1095	11.423	0.05 ± 0.00		0.10 ± 0.00	10.71 ± 0.02
trans-Sabinol	1137	1137	13.036	0.06 ± 0.00		0.14 ± 0.00	
Camphor	1143	1141	13.267	0.12 ± 0.00			3.72 ± 0.03
Isoborneol	1154	1155	13.787				1.04 ± 0.02
Borneol	1164	1165	14.24	0.14 ± 0.00			0.46 ± 0.01
Isononyl acetate	1171	1171	14.53				3.45 ± 0.01
Terpinen-4-ol	1180	1174	14.944	38.53 ± 0.04		0.95 ± 0.00	0.90 ± 0.02
α-Terpineol	1190	1186	15.34	2.16 ± 0.03		2.50 ± 0.01	2.00 ± 0.00
γ-Terpineol	1196	1199	15.606	0.21 ± 0.00			
Citronellol	1226	1223	16.923				2.50 ± 0.00
Geraniol	1254	1249	18.11				1.28 ± 0.00
Linalool acetate	1255	1254	18.194				9.60 ± 0.02
Bornyl acetate	1285	1287	19.562				0.21 ± 0.00
α-terpinyl acetate	1349	1346	22.374				10.93 ± 0.06
Neryl acetate	1364	1359	23.038				0.44 ± 0.00
Geranyl acetate	1384	1379	23.898				0.80 ± 0.02
α-Gurjunene	1409	1409	25.023			0.12 ± 0.00	
(E)-Caryophyllene	1420	1417	25.443	0.38 ± 0.01			1.80 ± 0.00
Aromadendrene	1439	1439	26.282			0.69 ± 0.01	
9-epi-Caryophyllene	1462	1464	27.225			0.17 ± 0.00	
Viridiflorene	1497	1496	28.693			0.07 ± 0.00	
Total peak area				564,685,150	117,582,225	142,637,552	98,030,240
Total of identified compounds (%)				99.76	98.12	99.91	74.53

^1^—Retention indices based on n-alkane series under identical experimental conditions and comparison was performed with the mass spectra library search NIST [33]; SD—standard deviation calculated for *n* (*n* = 3) GC–MSD analysis.

**Table 2:** Zone of inhibition of essential oils used in the study (mm).

Bacteria	Tea Tree	Rosemary	Eucalyptus	Lavender
*E. coli*	21			
*S. aureus*		13	13	13
*E. coli* ATCC 25922	18			
*S. aureus* ATCC 25923		13	13	13
*S. Typhi*	15		15	
*C. koseri*	13			

**Table 3:** Minimum inhibitory concentration (MIC); values of essential oils against bacteria (mg/mL) ^1^.

Bacteria	Tea Tree	Rosemary	Eucalyptus	Lavender
*E. coli*	3.1			
*S. aureus*				
*E. coli* ATCC 25922	2.7			
*S. aureus* ATCC 25923		1.4	2.9	2.1
*S. Typhi*	6.2			
*C. koseri*	3.4			

^1^—Values expressed the MIC as >the maximum concentration tested (50 mg/mL).

## Data Availability

Data is contained within the article.
